# Purinergic Signaling in Neuron-Astrocyte Interactions, Circadian Rhythms, and Alcohol Use Disorder

**DOI:** 10.3389/fphys.2018.00009

**Published:** 2018-02-06

**Authors:** Daniel Lindberg, Lindsey Andres-Beck, Yun-Fang Jia, Seungwoo Kang, Doo-Sup Choi

**Affiliations:** ^1^Neurobiology of Disease, Mayo Clinic College of Medicine, Rochester, MN, United States; ^2^Department of Molecular Pharmacology and Experimental Therapeutics, Mayo Clinic College of Medicine, Rochester, MN, United States; ^3^Department of Psychiatry and Psychology, Mayo Clinic College of Medicine, Rochester, MN, United States

**Keywords:** circadian, adenosine, glutamate, AUD, ethanol, astrocyte

## Abstract

Alcohol use disorder (AUD) is a debilitating condition marked by cyclic patterns of craving, use, and withdrawal. These pathological behaviors are mediated by multiple neurotransmitter systems utilizing glutamate, GABA, dopamine, ATP, and adenosine. In particular, purines such as ATP and adenosine have been demonstrated to alter the phase and function of the circadian clock and are reciprocally regulated by the clock itself. Importantly, chronic ethanol intake has been demonstrated to disrupt the molecular circadian clock and is associated with altered circadian patterns of activity and sleep. Moreover, ethanol has been demonstrated to disrupt purinergic signaling, while dysfunction of the purinergic system has been implicated in conditions of drug abuse such as AUD. In this review, we summarize our current knowledge regarding circadian disruption by ethanol, focusing on the reciprocal relationship that exists between oscillatory neurotransmission and the molecular circadian clock. In particular, we offer detailed explanations and hypotheses regarding the concerted regulation of purinergic signaling and circadian oscillations by neurons and astrocytes, and review the diverse mechanisms by which purinergic dysfuction may contribute to circadian disruption or alcohol abuse. Finally, we describe the mechanisms by which ethanol may disrupt or hijack endogenous circadian rhythms to induce the maladaptive behavioral patterns associated with AUD.

## Disruption of circadian rhythms in addictive behaviors

Time is an important factor governing human behavior. Each day we engage in repeated patterns of activity regarding work, sleep, and eating. Although artificial and cultural traditions may inform our daily schedules and activities, robust, intrinsic physiological rhythms heavily influence our thoughts and behaviors. These circadian rhythms are entrained by external lighting cues and are reciprocally modified by our behaviors and environment, which may alter the phase and amplitude of biological rhythms controlling sleep, consumption, and other behaviors (Partch et al., 2014). As such, disruption of circadian rhythms often results in maladaptive or pathologic behavior. Conversely, our behaviors and environment can adversely affect our circadian rhythms, producing maladaptive behavioral patterns that can contribute to substance abuse and alcohol use disorder (AUD) (Parekh et al., 2015).

**Figure 1:**
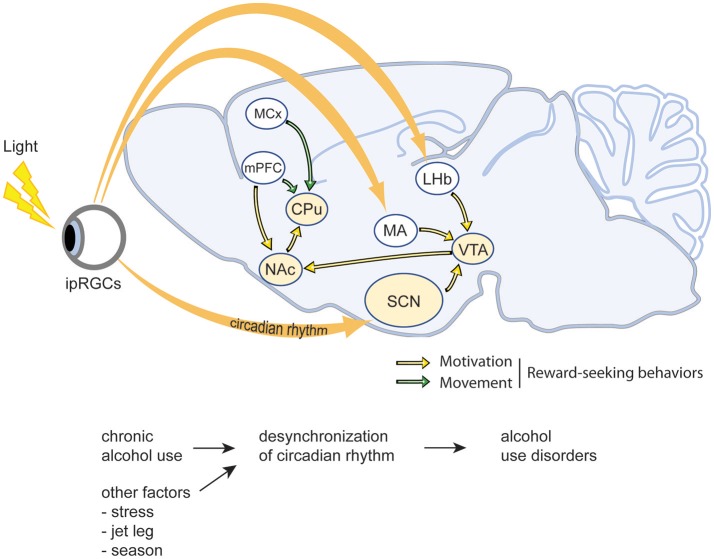
Schematic illustration of the interactions between circadian rhythms and reward-related brain regions. As the central pacemaker, the suprachiasmatic nucleus (SCN) receives photic input from the retina and thereby synchronizes internal rhythmicity with the 24 h light-dark cycle to regulate the circadian rhythms. The ventral tegmental area (VTA), nucleus accumbens (NAc), caudate putamen (CPu), as well as the medial prefrontal cortex (mPFC) and motor cortex (MCx) are the major regions involving in motivation and movement, thus regulate reward-seeking behaviors. ipRGCs, intrinsically photosensitive retinal ganglion cells; LHb, lateral habenula; SCN, suprachiasmatic nucleus; MA, medial amygdala; VTA, ventral tegmental area; mPFC, medial prefrontal cortex; NAc, nucleus accumbens; MCx, motor cortex; CPu, caudate-putamen.

At the molecular level, circadian rhythms are governed by auto-inhibitory transcriptional feedback loops mediated by the transcriptional activators *Clock* and *Bmal* and their inhibitory targets *Period 1 (Per1), Period 2 (Per2), Cryptochrome 1 (Cry1)*, and *Cryptochrome 2 (Cry2)* (Partch et al., 2014; Videnovic et al., 2014). More specifically, *Clock* and *Bmal* are transcriptional activators that induce the expression of *Period* and *Cryptochrome* proteins, which progressively accumulate throughout the circadian night and inhibit the activity of *Clock* and *Bmal* (Partch et al., 2014; Takahashi, 2015). Although this cycle is ubiquitous, and occurs in multiple cell types throughout the body, the mammalian suprachiasmatic nucleus (SCN) is the epicenter and central pacemaker of the mammalian circadian clock. As illustrated in Figure 1, the SCN receives photic input from the retina, which entrains molecular rhythms to environmental stimuli via the light-dependent degradation of *Cryptochrome* proteins, and subsequently conveys this information to distant regions of the CNS and periphery in order to synchronize independently functioning circadian clocks throughout the body (Partch et al., 2014; Takahashi, 2015). Interestingly, other environmental stimuli such as food, sex, and drugs can also influence the phase and amplitude of the circadian clock, both within the SCN as well as other regions of the brain more directly involved in behavior and reward (Rosenwasser, 2010; Albrecht, 2013; Damaggio and Gorman, 2014; Parekh et al., 2015).

Many humans live irregular lives that are incompatible with the natural cycles of the outside world. We work unnatural shifts, sleep at irregular hours for truncated or protracted periods of time, and eat with profound temporal indiscretion. These types of circadian disruptions can have tremendous impact on our intrinsic circadian clocks and adversely affect our health and behavior (Figure 1). Multiple studies indicate that circadian disruption caused by irregular shift work or repeated jetlag increases the risk of developing AUD (Trinkoff and Storr, 1998; Hasler et al., 2014). Similarly, work in animal models has demonstrated that circadian disruption via exposure to constant light or continuously variable light-dark cycles can increase ethanol drinking, promote relapse, and contribute to the development of AUD and mood disorders such as anxiety and depression (Rosenwasser and Fixaris, 2013; Rosenwasser et al., 2015). Genetic models disrupting the molecular circadian clock have revealed similar results. For example, Per2 mutant mice exhibit increased ethanol preference, and Per1 knockout animals fail to sensitize to cocaine and display increased conditioned place preference in response to drugs (Abarca et al., 2002; Spanagel et al., 2005a; Perreau-Lenz et al., 2009). In *Drosophila*, genetic disruption of the molecular circadian clock abolishes the development of ethanol tolerance and inhibits sensitization to the behavioral effects of cocaine (Andretic et al., 1999; Pohl et al., 2013). Alternatively, other studies have demonstrated that circadian disruption has little effect on ethanol intake. For example, Summa et al. showed that environmental or genetic disruption of circadian rhythms via 12 weeks of light/dark reversal or clock mutation, respectively, had little effect on ethanol intake in mice administered a liquid diet (Summa et al., 2013, 2015). This may be due to the methods utilized to measure ethanol intake or the lack of liquid choice imposed by such a restricted liquid diet. However, these studies also open the possibility that other factors related to circadian disruption as opposed to disruption of rhythms itself contribute to increased ethanol consumption.

Conversely, chronic alcohol abuse has been demonstrated to disrupt circadian rhythms (Rajakrishnan et al., 1999; Rosenwasser et al., 2005; Spanagel et al., 2005b; Kosobud et al., 2007; Ruby et al., 2009). Human patients suffering from AUD display diverse but markedly abnormal patterns of circadian activity, which may persist during abstinence and increase the risk of relapse (Brower, 2001). Furthermore, AUD has been associated with mutations and single-nucleotide polymorphisms (SNPs) in circadian genes including *Clock, Per1, Per2, and Per3* (Spanagel et al., 2005a; Kovanen et al., 2010; Sjöholm et al., 2010; Dong et al., 2011; Gamble et al., 2011; Blomeyer et al., 2013; Baranger et al., 2016). Although relatively few human studies have examined the effects of ethanol on the circadian clock, *Clock* mRNA expression was significantly lower in peripheral mononuclear cells isolated from AUD patients (Huang et al., 2010), and fibroblasts isolated from AUD patients exhibited Per2 cycles whose duration was inversely proportional to disease severity (Huang et al., 2010; McCarthy et al., 2013). In animal models, chronic ethanol administration disrupts photic entrainment of the SCN molecular clock and alters circadian patterns of locomotion (Ruby et al., 2009). Other studies have demonstrated that ethanol shortens the free-running period of mice in constant darkness, supporting the findings of truncated Per2 cycles in human patients with severe AUD (Seggio et al., 2009). These behavioral changes may be associated with ethanol-mediated modulation of the molecular circadian clock. Gene expression analysis of the nucleus accumbens revealed that chronic ethanol administration increases the expression of multiple components of the circadian clock, including *Bmal*, and *Per3* (Melendez et al., 2012). Others have shown that chronic ethanol administration via a two-bottle choice paradigm, decreases *Clock* expression in both the nucleus accumbens and the VTA (Ozburn et al., 2013). In the SCN, ethanol has been demonstrated to alter the phase of *Per2* and *Per3* rhythms, perhaps providing some explanation for ethanol-mediated disruption of circadian patterns of sleep and activity (Chen et al., 2004). Thus, ethanol has been demonstrated to alter circadian rhythms of gene expression and behavior, exerting combinatorial affects on both the central pacemaker of the SCN as well as peripheral sites involved in behavior and reward. Although these effects have been widely reported, it is possible that the circadian disrupting effects of ethanol are dependent upon the dose or time of ethanol exposure. For example, in human patients, administration of a single dose of alcohol does not significantly alter the circadian phase or amplitude (Burgess et al., 2016). Alternatively, chronic ethanol administration greatly affects circadian rhythms (Rajakrishnan et al., 1999; Rosenwasser et al., 2005; Spanagel et al., 2005b; Kosobud et al., 2007; Ruby et al., 2009). Furthermore, ethanol has been demonstrated to exert increased sedative effects when consumed during the circadian night, perhaps exacerbating the circadian disrupting effects of even small doses of alcohol (Walsh et al., 1991).

Circadian clock genes are expressed in regions of the brain associated with reward, including the nucleus accumbens, caudate putamen, medial prefrontal cortex, and ventral tegmental area (Logan et al., 2014; Parekh et al., 2015). Although these regions are dependent on the SCN to synchronize the circadian oscillations of individual cells, these molecular cycles may be out of phase with the SCN, owing to their differential sensitivity to light-independent external stimuli, including drugs of abuse (Logan et al., 2014). For example, ethanol, cocaine, methamphetamine, and nicotine have been shown to induce anticipatory behavior and entrain Per1 expression rhythms in the nucleus accumbens, prefrontal cortex, and amygdala (Kosobud et al., 1998, 2007; White et al., 2000; Gillman et al., 2008). Other studies have shown that chronic ethanol intake via a restrictive liquid diet alters the phase of *Per1* rhythms in the arcuate nucleus, which sends projections to the nucleus accumbens and is heavily involved in consummatory behavior (Chen et al., 2004). Importantly, chronic ethanol intake has been demonstrated to reduce the expression of *Clock* within the ventral tegmental area (Ozburn et al., 2013; Parekh et al., 2015). Conversely, RNAi-mediated knockdown of *Clock* expression within the VTA increases ethanol intake (Roybal et al., 2007; Mukherjee et al., 2010). Furthermore, *Clock* mutant mice exhibit increased locomotor activity, reduced anxiety-like and depression-like behaviors, and more frequent intracranial self-stimulation at a lower threshold (McClung et al., 2005; Roybal et al., 2007). These behaviors are associated with an increase in dopaminergic activity in the VTA and a general increase in glutamatergic tone, suggesting that circadian disruption may induce both region-specific and global changes in synaptic signaling which may further desynchronize circadian rhythms and contribute to the behavioral manifestations of AUD (McClung et al., 2005; Beaule et al., 2009).

Together, these results suggest that circadian rhythms and reward-regulated behaviors exist in a reciprocal relationship wherein molecular circadian oscillations dictate rhythms of anticipation and reward seeking, which may be re-entrained by ethanol and drugs of abuse. Oscillations of the circadian clock may help to establish behavioral rhythms of reward by maintaining a fluctuating baseline of anticipation against which the hedonic value of rewarding stimuli may be judged. Drugs of abuse may coopt this system by altering the phase, frequency, or amplitude of endogenous circadian rhythms, prompting maladaptive manifestations such as craving and drug seeking behaviors. These drug-induced alterations of circadian cycles may occur not only at the level of the molecular circadian clock but also affect the rhythmicity of multiple neurotransmitter systems utilizing glutamate, dopamine, and adenosine.

## Purinergic signaling and circadian rhythms

**Figure 2:**
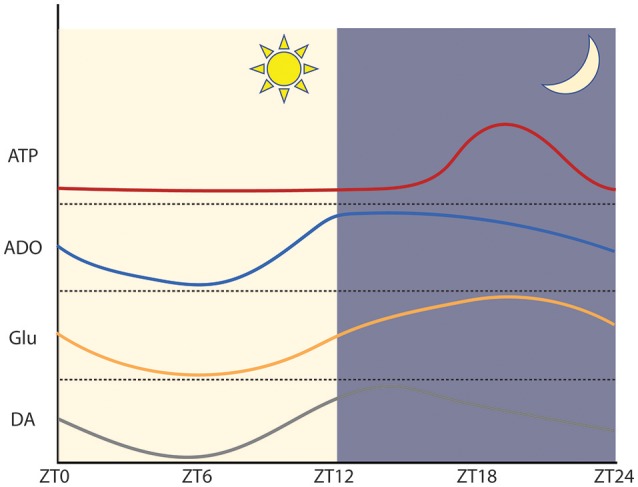
Approximation of the phase and amplitude of circadian neurotransmitter oscillations within regions of abundance in mice. Within the SCN ATP and glutamate peak during the circadian night and reach a trough during the circadian day. In the SCN, adenosine peaks early during the circadian night. Within the dorsal striatum, dopamine peaks early during the circadian night. ATP: adapted from Womac et al. (2009) and Marpegan et al. (2011). Adenosine: adapted from Chagoya de Sánchez (1995). Glutamate: adapted from Brancaccio et al. (2017). Dopamine: adapted from Ferris et al. (2014).

Each night, we abate the bustling activities of daytime and retire to our beds, where we sleep until awakening the following morning. Cyclic, circadian behaviors such as these are necessarily driven by periodic physiological processes, most often governed by autonomous, regularly-occurring negative feedback loops. At the molecular level, circadian timing is regulated by oscillatory transcription and translation of auto-inhibitory clock genes (Partch et al., 2014; Takahashi, 2015). As shown in Figure 2, the molecular periodicity regulates innumerable neurological processes including neuronal excitability, glutamatergic transmission, dopamine signaling, neuronal and glial energy homeostasis, and ATP and adenosine handling (Bass and Takahashi, 2010; Ruby et al., 2014a; Parekh et al., 2015). In turn, functional fluctuations of these cellular, bioenergetic, and signaling processes manifest as behavioral rhythms that permeate our everyday life.

Purinergic signaling, mediated by adenosine and ATP, is a powerful regulator of sleep that reciprocally regulates circadian gene expression (Ruby et al., 2014a; Urry and Landolt, 2015; Reichert et al., 2016). ATP is the primary biochemical energy carrier, and is also utilized as a neurotransmitter at glutamatergic, GABAergic, dopaminergic, and adrenergic synapses, where it is co-secreted into the synaptic cleft by the presynaptic neuron or released via calcium-dependent exocytosis by astrocytes (Burnstock, 2007, 2008; Lindberg et al., 2015). Here, ATP modulates synaptic activity according to the subtypes of purinergic receptors expressed by neighboring neurons and astrocytes. ATP and its phosphate-bound hydrolysis products ADP and AMP activate P2 receptors, which are predominantly localized to the post-synaptic membrane as well as on astrocytes, microglia, and oligodendrocytes (Burnstock, 2007). P2 receptors are subdivided into P2X and P2Y subtypes; P2X receptors are ligand-gated ion channels with variable permeability to Na^+^, K^+^, and Ca^2+^, while P2Y receptors are GPCRs coupled to G_s_, G_i/o_, or G_q/11_ (Burnstock, 2007; Lindberg et al., 2015).

Interestingly, concentrations of extracellular ATP exhibit marked circadian rhythmicity within the SCN, peaking during the latter half of the dark period (subjective night) and reaching a trough during the subjective day (Womac et al., 2009; Marpegan et al., 2011). Numerous metabolic processes including glycolysis, gluconeogenesis, and fatty acid oxidation are transcriptionally regulated by molecular components of the circadian clock, which bind to multiple regulatory elements effecting the expression of enzymes that carry out the rate-limiting steps of metabolic processes (Mazzoccoli et al., 2012; Eckel-Mahan and Sassone-Corsi, 2013). For example, the mammalian SCN displays circadian oscillations in the expression of hexose kinase 1 (HK1), which regulates the concentration of useable glucose, as well as several mitochondrial mediators of oxidative phosphorylation (Mazzoccoli et al., 2012). This temporal control of energy homeostasis may impose circadian limitations on the availability of ancillary ATP utilizable for vesicular transport and synaptic signaling, thus contributing to the circadian rhythmicity of synaptic ATP. This is supported by the observation that SCN astrocytes display circadian mitochondrial calcium rhythms which mirror the extracellular accumulation of ATP and are necessary for the nocturnal peak in synaptic ATP (Burkeen et al., 2011).

Alternatively, rhythmic circadian accumulation of synaptic ATP may be a consequence of intrinsic circadian rhythms governing neuronal and astrocytic excitability and activity. Both neurons and astrocytes within the SCN exhibit robust rhythmic circadian activity demonstrable by voltage indicators and calcium sensors (Brancaccio et al., 2017). These cycles are anti-phasic, with neuronal activity peaking during mid-circadian daytime and astrocytes displaying peak calcium concentrations during the circadian night (Brancaccio et al., 2017). Interestingly, extracellular concentrations of SCN glutamate display circadian oscillations that are synchronous with astrocytic calcium rhythms and anti-phasic to patterns of neuronal activity (Brancaccio et al., 2017). These extracellular glutamate rhythms appear to approximate the phase of synaptic ATP concentrations, suggesting that vesicular release of glutamate and ATP may be orchestrated by astrocytes and act to inhibit neuronal activity during the circadian night. Additionally, rhythmic cycles of extracellular ATP may help to set the gain or reinforce the phase of the molecular circadian clock.

Although clock-mediated control of metabolism, ATP synthesis, and purine and glutamate release may mediate the rhythmic oscillations of extracellular ATP, numerous investigations suggest that ATP may reciprocally regulate the activity of the molecular circadian clock. For example, exposure of murine microglia to ATP transiently induces Per1 expression in a manner sensitive to P2X7 antagonism (Nakazato et al., 2011). Furthermore, both the hypothalamus and somatosensory cortex exhibit circadian variation in the expression of P2X7 receptors, which is increased during the circadian day and decreased at night (Krueger et al., 2010). Interestingly, hypothalamic P2X7 expression is also induced by sleep deprivation (Krueger et al., 2010). Together, these results suggest that enhanced diurnal ATP signaling via P2X7 may partially contribute to the steady rise of Per1 expression during the circadian daytime. Perhaps more accurately, oscillatory P2X7 signaling may help control the gain or amplitude of the molecular clock, producing more dramatic variation of clock gene expression than would be possible in the absence of extracellular ATP.

Like ATP, adenosine has also been demonstrated to reciprocally regulate circadian rhythms (Ruby et al., 2014a; Urry and Landolt, 2015; Reichert et al., 2016). Although adenosine may be synthesized *de novo* from activated ribose (PRPP) in anabolic reactions requiring amino acids, it is more commonly derived from catabolism of high-energy ATP produced by oxidative phosphorylation (Lindberg et al., 2015). Synaptic ATP secreted via calcium-dependent exocytosis by neurons and astrocytes is quickly converted to adenosine by a family of exonucleotidases (Burnstock, 2007). Likewise, additional adenosine can be packaged into vesicles following intracellular ATP degradation or be transported across the membrane by nucleoside transporters such equilibrative nucleoside transporter 1 (ENT1) located on neurons and astrocytes (Burnstock, 2007). Extracellular adenosine acts upon P1 receptors, which are predominantly localized to the presynaptic membrane as well as on astrocytes, microglia, and oligodendrocytes (Burnstock, 2008). The four subtypes of P1 receptors (A1, A2A, A2B, and A3) activate varying subsets of G_s_, G_i/o_, and G_q/11_ to exert diverse effects on cellular and CNS function (Burnstock, 2007, 2008). A1 receptors bind adenosine to activate G_i/o_ and inhibit cAMP production, while A2A and A2B receptors (A2AR and A2BR) increase cAMP via G_s_ (Burnstock, 2007). A3 may activate either G_i/o_ or G_q/11_, resulting in decreased cellular cAMP and increased concentrations of intracellular inosine-1, 4, 5-triphosphate (IP3) (Burnstock, 2007). Importantly, the stepwise degradation of ATP to ADP, AMP, and finally adenosine produce a diverse and constantly varying array of ligands that may act upon both P1 and P2 receptors to exert a repertoire of cellular and signaling effects that may vary depending upon the relative concentration of each purine.

Similar to ATP, adenosine undergoes circadian oscillations of synaptic and extracellular concentrations (Chagoya de Sánchez, 1995; Porkka-Heiskanen et al., 1997, 2000). Interestingly, levels of extracellular adenosine peak early during circadian nighttime and progressively increase throughout the course of the circadian day (Porkka-Heiskanen et al., 1997). This circadian rhythm is synchronized with the cyclic expression of transporters responsible for regulating synaptic adenosine, including ENT1 (Ruby et al., 2014b). Interestingly, disruption of ENT1 function via genetic knockout reduces synaptic adenosine concentrations and lowers the peak expression of clock genes, including Per2 (Ruby et al., 2014b). Furthermore, ENT1 KO mice display altered circadian rhythms marked by early onset of an elongated and hyperactive active phase (Ruby et al., 2014b). Similar circadian disruptions are also induced by caffeine and A2AR antagonism, which lengthen the period of molecular clock oscillations and induce behavioral changes in the sleep-wake cycle (Antle et al., 2001; Burke et al., 2015). This suggests that adenosine signaling may not only regulate the amplitude of circadian oscillations but also modulate the phase of the circadian clock.

Perhaps the most well-established behavioral role of adenosine is the exertion of “sleep pressure.” As previously described, adenosine accumulates in the extracellular environment during the daytime, peaking early during the circadian night and progressively declining during the circadian sleep phase. Sleep deprivation elevates extracellular levels of adenosine and produces a phase shift in the sleep-wake cycle, transiently lengthening the period of the sleep cycle and increasing the duration of slow wave sleep in a manner proportional to the duration of sleep deprivation (Porkka-Heiskanen et al., 1997; Wurts and Edgar, 2000). These results suggest that extracellular adenosine acts to promote the drive to sleep, continuously increasing in extracellular concentration until resolved by the induction of sleep.

**Figure 3:**
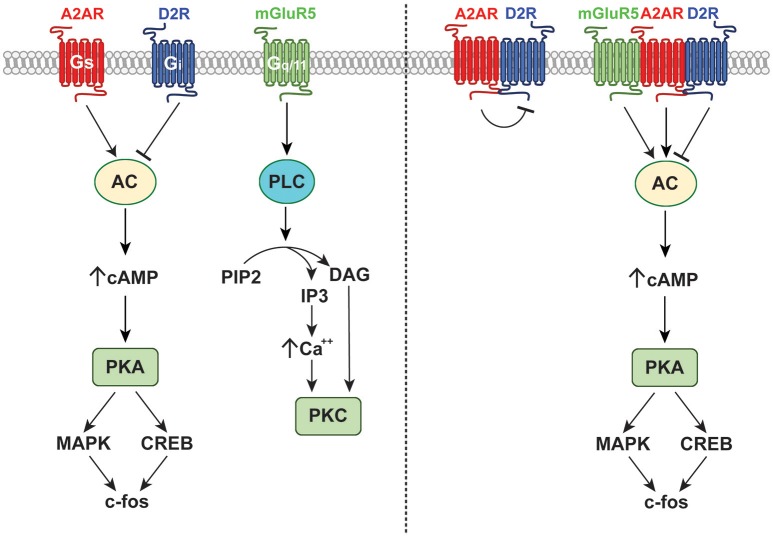
Receptor and signaling interactions of the A2AR. The A2AR forms functional A2A-D2 receptor dimers as well as mGluR5-A2A-D2 receptor heteromers. These interactions affect binding with endogenous ligands and modulate downstream signaling events as shown.

Importantly, the somnogenic and circadian effects of adenosine are at least partially mediated by interactions with the dopaminergic and glutamatergic neurotransmitter systems. As previously described, astrocytes control circadian timekeeping in the SCN by regulating extracellular levels of glutamate (Brancaccio et al., 2017). This is associated with circadian fluctuations in glutamate uptake as well as diurnal cycles in the activity of glutamine synthetase (GS), which converts glutamate into glutamine (Leone et al., 2015). Interestingly, several regions of the brain including the cortex, nucleus accumbens, amygdala, and caudate putamen demonstrate cyclic expression of the metabotropic glutamate receptor 5 (mGluR5) (Elmenhorst et al., 2016). Furthermore, mGluR5 knockout animals exhibit altered sleep-wake architecture, marked by reduced REM sleep time during the light phase with shorter bouts of REM sleep and reduced state transitions in the non-REM sleep-REM cycle (Ahnaou et al., 2015). Moreover, these animals exhibited reduced slow wave activity and sleep drive after sleep deprivation, demonstrating that like adenosine, glutamate and mGluR5 are important for regulating the phase and amplitude of the sleep-wake cycle (Ahnaou et al., 2015). Importantly, mGluR5 has been demonstrated to form heterodimers with the A2AR within the striatum (Figure 3), where complex interactions between adenosine and glutamate signaling and cyclic circadian oscillations in purinergic and glutamatergic signaling modulate behaviors related to motivation and reward (Ferré et al., 2002; Brown et al., 2012). Other studies have demonstrated that both the type 2 and 3 metabotropic glutamate receptors (GluR2 and GluR3) are also important for regulating the circadian sleep cycle. For example, double knockout animals exhibit altered activity cycles, marked by a decrease in immobility-determined sleep time, increased sleep fragmentation, and heightened sensitivity to the circadian effects of light (Pritchett et al., 2015). Given that adenosine has been demonstrated to gate glutamatergic photic input into the SCN and modulate non-photic circadian cycles of glutamatergic and non-glutamatergic transmission controlling sleep and activity, it is possible that adenosine acts to refine the relative sensitivity to numerous convergent neurotransmitter systems and that multiple different neurotransmitters act in concert to set the phase and amplitude of circadian rhythms.

A 2015 report by Morioka and colleagues partially demonstrated the complex interactions of multiple neurotransmitter systems in influencing the molecular clock. Simultaneous treatment of cultured astrocytes with glutamate, serotonin, and dopamine led to induction of *Per1* expression and a delayed increase of *Bmal1* expression and a decrease of *Cry1* expression (Morioka et al., 2015). In contrast, individual treatment with any one of these transmitters led only to a transient increase in Per1 expression, which was not maintained through further modulation of the molecular clock (Morioka et al., 2015). These results demonstrate the complex neurotransmitter interactions that govern circadian rhythms and demonstrate that dopamine may also play an important function in circadian control. Other studies have extended this finding, suggesting that the dopaminergic system is reciprocally regulated by the circadian clock and also mediates some cyclic circadian behaviors. This is exemplified by patients suffering from Parkinsonian dopamine deficiency, who often present with altered sleep-wake cycles and impaired entrainment to external lighting cues (Videnovic and Golombek, 2013). Like most neurotransmitters, dopamine undergoes circadian oscillations in extracellular abundance, peaking during the middle of the circadian night and reaching a trough during the circadian day (Ferris et al., 2014). Interestingly, these rhythms are in phase with circadian cycles of both the dopamine D2 receptor (D2R) and A2AR within the caudate putamen (Figure 3; Weber et al., 2004). Dopamine rhythms within regions of the brain related to reward and goal-directed behavior, including the nucleus accumbens and caudate putamen, mediate behavioral rhythms such as food anticipation and reward seeking behavior (Gallardo et al., 2014; Parekh et al., 2015). Moreover, rewarding environmental stimuli can entrain circadian dopamine rhythms and produce parallel phase changes in clock gene expression and extracellular adenosine (Kosobud et al., 2007; Angeles-Castellanos et al., 2008; Gillman et al., 2008). Together, these findings support the concept that dopamine, adenosine, and the circadian clock exist in a reciprocal relationship that coordinates reward-based consummatory behaviors, which may be altered by pathologic dysfunction such as substance use disorder (SUD) and AUD.

Numerous studies have demonstrated that adenosine and dopamine receptors form heterodimers that alter the activity and/or affinity of both receptors (Ferré et al., 2007). Within the striatum, dopamine D1 receptors (D1Rs) form heterodimers with adenosine A1 receptors (A1Rs), resulting in impaired D1R signaling (Ginés et al., 2000). This heterodimerization is promoted by A1R agonists, which impair D1R-mediated increases in cAMP, and is prevented by pretreatment with D1R agonists (Ginés et al., 2000). These results suggest that the precise phase relationship of circadian adenosine and dopamine oscillations may dictate the precise response to either transmitter, and may permit changes in downstream signaling events without altering absolute neurotransmitter levels. Like A1R and D1R, dopamine D2R form heterodimers with adenosine A2AR, resulting in allosteric antagonism upon activation or inhibition of the purinergic receptor (Ferre et al., 1991; Dasgupta et al., 1996). Specifically, A2AR agonists and antagonists inhibit D2R activation, minimizing G_i_ signaling and promoting the activation of inhibitory GABAergic medium spiny neurons that affect action selection and reward-based decision making (Ferré et al., 1993; Strömberg et al., 2000). Conversely, D2R signaling via G_i_ directly counteracts A2AR-mediated increases in cAMP driven by G_s_ activation (Kull et al., 1999; Hillion et al., 2002). Therefore, given that A2AR and D2Rs undergo circadian oscillations, it is possible that shifting the phase of any one of these rhythms could drastically alter synaptic responses to multiple endogenous ligands or external stimuli in a manner dependent upon the current ratio of A2AR to D2R. Additional complexity arises from the fact that stimulation of GluR5 receptors potentiates the antagonistic effect of A2AR on D2R binding, suggesting the existence of mGluR5-A2A-D2 receptor heteromers (Popoli et al., 2001; Ferré et al., 2002). These complex receptors may mediate some synergistic actions of A2AR and mGluR5, including mGluR5R-mediated potentiation of A2AR signaling via MAPK and PKA with phosphorylation of dopamine- and cAMP-regulated neuronal phosphoprotein 32 (DARPP-32) (Popoli et al., 2001; Nishi et al., 2003). This may allow co-stimulation with glutamate and adenosine to successfully overcome tonic D2R-mediated inhibition of the indirect striatal pathway to induce activity-dependent changes in gene expression. In fact, central coadministration of selective A2AR and mGluR5 agonists induces an increase in the striatal expression of *c-fos*, which was unachievable by individual activation of either receptor type (Popoli et al., 2001; Figure 3).

Taken together, these findings demonstrate that multiple neural pathways and neurotransmitter systems converge to affect the molecular circadian clock and mediate rhythmic circadian behaviors. Under normal physiological conditions or mild external assault, this extraordinary convergence may underlie the tremendous stability and fidelity of the circadian system. However, this confluence of signaling pathways also provides an abundance of entry points by which external stimuli may modulate or adversely affect the circadian clock. Adenosine and ATP appear relatively unique in their nearly ubiquitous role of modulating other neurotransmitter systems that provide input into the molecular clock. As such, it is possible that purinergic signaling modulates the gain of individual clock inputs and minimizes the damage induced by disruption of any single neurotransmitter system. Conversely, ethanol and other drugs of abuse that adversely affect the purinergic system may induce dysfunction in disparate signaling pathways. Similarly, the circadian clock exerts divergent effects on dopaminergic, glutamatergic, and purinergic signaling pathways. Although this serves as an effective means to amplify and convey circadian signals, pathological disruption of the clock may be exaggerated and perpetuated by maladaptive downstream events. Likewise, this divergence may allow circadian disruption to affect many behaviors mediated by glutamatergic and dopaminergic signaling but only tangentially related to the circadian clock. Importantly, these neurotransmitter systems as well as the molecular clock itself are coordinated by the activity of both neurons and astrocytes, which directly contribute to circadian synaptic signaling and may be involved in the pathophysiology of AUD.

## Neuron-astrocyte interactions in the regulation of circadian rhythms and ethanol response

Although we typically consider synaptic transmission in terms of its presynaptic and post-synaptic neuronal components, a third player is critically important to neuromodulation and synaptic signaling. Astrocytes are multifunctional cells that play a significant role in neurogenesis, bioenergetics, neurotransmission, immune response, amino acid neurotransmitter clearance, and ionic homeostasis (Ransom and Ransom, 2012). These glial cells are 10 times more abundant than neurons within the CNS and have heterogeneous morphology and gene expression profiles dependent upon their location, neuronal contacts, and overall microenvironment (Pannasch and Rouach, 2013; Heller and Rusakov, 2015). This variability enables astrocytes to function effectively in a variety of roles throughout the brain. Synaptic transmission is regulated by a “tripartite synapse” consisting of a presynaptic neuron, post-synaptic neuron, and perisynaptic astrocyte (Araque et al., 1999). Astrocytic processes in close proximity to the synaptic milieu remove glutamate and other neurotransmitters that spill over from the synaptic cleft, protecting neurons from excitotoxicity, and inducing a wide variety of gliotransmission events that may release glutamate, ATP, adenosine, and other signaling molecules (Peters et al., 1991; Diamond, 2005; Rusakov et al., 2011; Ransom and Ransom, 2012). Furthermore, recent evidence suggests that astrocytes are at least partially responsible for regulating molecular circadian rhythms both within the SCN as well as within other regions of the CNS more directly involved in behavioral output (Jackson, 2011; Marpegan et al., 2011; Brancaccio et al., 2017). Importantly, ethanol has also been demonstrated to modulate both neuron and astrocyte-mediated purinergic and glutamatergic signaling (Rossetti and Carboni, 1995; Dahchour and De Witte, 1999; Choi et al., 2004; Nam et al., 2011; Wu et al., 2011). Thus, accounting for astrocytic influence on neurotransmission may permit a more comprehensive understanding of the synaptic and extrasynaptic signaling events that contribute to circadian disruption and neuropsychiatric conditions such as AUD.

As previously explained, long-term imaging studies revealed the presence of circadian oscillations of intracellular calcium in astrocytes, peaking during the circadian night and reaching a trough during daytime (Brancaccio et al., 2017). These calcium oscillations were found to be in phase with circadian rhythms of extracellular glutamate, which were abolished following pharmacological inhibition of astrocytic glutamate catabolism or genetic ablation of astrocytes (Brancaccio et al., 2017). Moreover, disruption of astrocytic glutamate release altered neuronal Per2 expression and adversely affected circadian rhythmicity within the SCN (Brancaccio et al., 2017). Conversely, glutamate uptake and mRNA levels of the glutamate-aspartate transporter (GLAST) were significantly reduced in both *Clock* and *Per2* mutant astrocytes (Beaule et al., 2009). Interestingly, astrocytes also undergo circadian oscillations of ATP release, the phase of which approximates the phase of astrocyte-mediated glutamate release (Womac et al., 2009; Burkeen et al., 2011; Marpegan et al., 2011). These ATP cycles are dependent upon the circadian clock and are abolished by mutation of the *Clock* or *Per2* genes (Marpegan et al., 2011). Other studies found that astrocytic deletion of the clock component *Bmal* lengthened the period of the neuronal molecular clock and induced parallel changes in the free-running period of mutant mice (Tso et al., 2017). These studies indicate that astrocytes and the astrocytic circadian clock are instrumental for regulating the neuronal circadian clock and may mediate some forms of circadian behavior. Moreover, astrocytes are intricately involved in the regulation of glutamatergic and purinergic neurotransmission, which converge to regulate and are reciprocally regulated by the circadian clock.

Although some glial cells may release glutamate into the synaptic environment via calcium-dependent gliotransmission, astrocytes primarily regulate glutamatergic transmission by clearing glutamate from the synaptic cleft (Ransom and Ransom, 2012). Approximately 90% of extracellular glutamate is taken up by the type 2 excitatory amino acid transporter (EAAT2; known as GLT1 in rodents), which is primarily expressed on the astrocytic plasma membrane (Bergles and Jahr, 1997; Rusakov et al., 2011). EAATs can transport both glutamate and aspartate. When active, they exchange one glutamate molecule as well as three sodium ions and one proton for a single ion of potassium (Yernool et al., 2004). As the primary EAAT subtype expressed in the striatum, EAAT2 has become a transporter of interest in the field of addiction research, especially since changes in EAAT2 surface expression have been shown to influence neurotransmission (Murphy-Royal et al., 2015). Following EAAT2-mediated synaptic clearance, glutamate is converted to glutamine by glutamine synthetase, which is primarily expressed in astrocytic processes that abut glutamatergic synapses (Derouiche and Frotscher, 1991). This glutamine is subsequently released from astrocytes and taken back into presynaptic neurons, where it is converted back to glutamate and repackaged into synaptic vesicles (Coulter and Eid, 2012). Alternatively, astrocytic glutamate may be packaged into vesicles and released by astrocytes as a synaptic gliotransmitter, or be utilized as a bioenergetic substrate of the TCA cycle following conversion to alpha-ketoglutarate by glutamate dehydrogenase (Ransom and Ransom, 2012). Importantly, both glutamate uptake and GS activity display circadian variability within the SCN, peaking during the light phase and reaching a trough during the circadian night (Leone et al., 2015). Moreover, ethanol has been demonstrated to induce the expression of EAAT1 and EAAT2 in organotypic cortical slice cultures (Zink et al., 2004). This suggests that the normal circadian variation in glutamate uptake and GS activity, which may modulate the pool of astrocytic glutamate and thereby control the concentration of glutamate within astrocytes as well as the relative proportion utilized for gliotransmission, energy production, and neuronal glutamine supply, may be disrupted or altered by ethanol. Such disruption may result in altered glutamatergic signaling or dysfunctional circadian glutamate rhythms, which may contribute to the behavioral manifestations of AUD.

Astrocytic regulation of extracellular glutamate is critical for the maintenance of synaptic function. Acutely, ethanol affects GABAergic and glutamatergic neurotransmission. As the primary inhibitory neurotransmitter in the brain, GABA typically inhibits the firing of other neurons, preventing the release of their stored transmitter pools. Acute ethanol exposure potentiates GABAergic transmission and directly alters glutamatergic signaling by inhibiting the activity of NMDA receptors (Ferre and O'brien, 2011). In alcohol withdrawal syndrome (AWS) there is a rebound from suppressed glutamatergic activity and a disruption of glutamate release regulation, resulting in increased extracellular glutamate and increased potential for excitotoxicity. For example, during alcohol withdrawal, extracellular glutamate levels are increased in the striatum and hippocampus (Rossetti and Carboni, 1995; Dahchour and De Witte, 1999). Furthermore, increased extracellular glutamate in the nucleus accumbens has been shown to increase ethanol drinking in ethanol dependent mice (Griffin et al., 2014). Although not circadian in nature, this creates a maladaptive cycle of glutamatergic unbalance and behavioral resolution. More specifically, ethanol withdrawal induces a hyperglutamatergic state that is resolved by ethanol drinking, which suppresses glutamate release by enhancing GABAergic and suppressing glutamatergic signaling until ethanol is metabolized and withdrawal returns.

**Figure 4:**
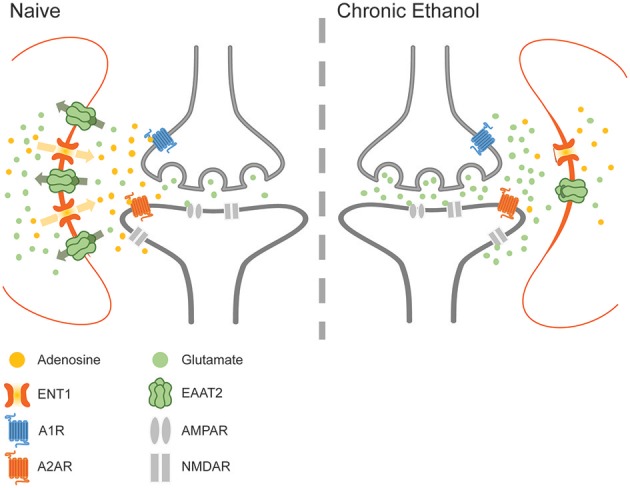
The effects of chronic ethanol exposure on ENT1-mediated adenosine signaling. Ethanol downregulates ENT1 and decreases adenosine in the synaptic cleft, impairing adenosine-mediated inhibition of glutamate release and increasing the concentration of glutamate within the synaptic environment during withdrawal.

Adenosine is an important regulator of glutamate signaling in both acute and chronic ethanol exposure (Lindberg et al., 2015). Astrocytes are a notable source of extracellular adenosine, which may regulate transporter-mediated adenosine release from both astrocytes and neurons (Figure 4). Furthermore, adenosine signaling via A1Rs has been shown to alter the expression and activity of EAAT2 and to influence ethanol consumption via downstream effects on NMDA receptors and ENT1 (Nam et al., 2011; Wu et al., 2011). Interestingly, inhibition or downregulation of ENT1 reduces the expression of EAAT2, while ENT1 overexpression increases the expression of EAAT2 and enhances glutamate uptake (Wu et al., 2010). Astrocytes abundantly express ENT1, which facilitates the diffusion of nucleosides across the plasma membrane. Although acute ethanol exposure inhibits ENT1-mediated adenosine uptake, chronic ethanol exposure down-regulates ENT1 expression and impairs nucleoside transport (Asatryan et al., 2011). Importantly, ENT1 null mice exhibit increased ethanol preference as well as reduced ethanol-induced ataxia and diminished hypnotic effects (Choi et al., 2004). These results suggest that purinergic and glutamatergic signaling mechanisms converge to control drinking behavior, perhaps by modulating the sensitivity to the intoxicating effects of ethanol. Therefore, as important regulators of purinergic signaling, astrocytes are uniquely poised to modulate multiple pathways of neurotransmission. Furthermore, astrocytic modulation of rhythmic synaptic transmission and purinergic modulation suggests that these cells may play an important role in the development and manifestation of addiction and withdrawal.

## Clinical perspectives and conclusion

Substance use disorder (SUD) can be viewed as robust maladaptive cycles of use and withdrawal. By disrupting circadian oscillations of neurotransmission and astrocytic modulation, drugs of abuse may hijack the powerful endogenous rhythms that mediate our daily thoughts and actions. Moreover, ethanol may induce novel persistent oscillations of GABAergic, dopaminergic, glutamatergic, and purinergic neurotransmission, which contribute to the intermittent craving, anticipation, and impulsivity underlying AUD. As such, effective pharmacological interventions for AUD may help to bolster or reset natural circadian rhythms in order to more effectively compete with the aberrant artificial oscillations induced by ethanol. Alternatively, these interventions could specifically disrupt ethanol-induced rhythms or incite global disruption of oscillatory transmission in the hopes that natural rhythms will be restored when the cycles resume.

Current treatment options for AUD are limited, and span the spectrum from archaic to experimental. Disulfiram, sold under the trade name Antabuse, was first approved by the FDA more than 50 years ago (Suh et al., 2006). Its pharmacologic efficacy is based upon its inhibition of the acetaldehyde dehydrogenase enzyme, resulting in the accumulation of the emetic acetaldehyde (Brewer, 1984). Accordingly, the neural mechanisms underlying disulfiram treatment may be similar to those responsible for aversion therapy. Namely, oscillating anticipatory cycles mediated by glutamate and dopamine may be disrupted and replaced by a novel negative association, which blunts the exaggerated amplitude of neurotransmitter rhythms and thereby facilitates their normalization. Alternatively, some studies have demonstrated that disulfiram may also be effective in treating cocaine addiction through off-target inhibition of dopamine-beta-hydroxylase, increasing the ratio of dopaminergic to noradrenergic tone and effectively attenuating drug-primed reinstatement of cocaine seeking (Schroeder et al., 2010).

Like disulfiram, naltrexone acts to reduce the reinforcing properties of ethanol and other drugs of abuse (Gianoulakis et al., 1996). In the CNS, naltrexone acts as an antagonist to mu and kappa opioid receptors, and is known to modulate both dopaminergic and glutamatergic signaling within the mesolimbic pathway (Spanagel et al., 1992; Lee et al., 2005; Chartoff and Connery, 2014). Interestingly, some studies have demonstrated that naltrexone may also upregulate the expression of A1Rs (Bailey et al., 2003). This suggests that naltrexone and other opioid receptor antagonists may effectively modulate the phase or amplitude of glutamatergic, dopaminergic, and purinergic oscillations in the striatum. Such phasic realignment may alter the neurological response to rewarding stimuli and reduce the neurological drive to drink.

Acamprosate is a synthetic analog of the endogenous amino acid taurine (Dahchour and De Witte, 2000). Although its precise mechanism of action is unknown, acamprosate has been demonstrated to alter GABAergic transmission as well as modulate the activity of the NMDA receptor (Dahchour and De Witte, 2000). Acamprosate is known to reduce withdrawal-induced excitotoxicity, and may also affect the action other neurotransmitters such as dopamine (Dahchour and De Witte, 2000).

Topiramate is an anticonvulsant medication that has demonstrated some efficacy for the treatment of AUD (Kenna et al., 2009). This sulfate-substituted monosaccharide is thought to primarily act by modulating the action of voltage-gated sodium channels, but has also been demonstrated to affect high-voltage activated calcium channels, GABA-A receptors, and AMPA receptors in the CNS (Johnson and Ait-Daoud, 2010). These channels and receptors are instrumental for the function of all neurons, and may cause diverse effects on multiple neural circuits, potentially correcting neurotransmitter imbalances or maladaptive phasic interference between different oscillatory signaling systems.

A common theme among AUD treatments is the modulation of multiple neurotransmitter systems at both the neuronal and astrocytic levels. This is the case even for purely experimental treatments such as ceftriaxone and fibrates, which may alter transcriptional regulation of multiple proteins involved in neurotransmission and synaptic plasticity (Sari et al., 2011; Abulseoud et al., 2014; Ferguson et al., 2014). As discussed, chronic alcohol abuse broadly disrupts synaptic signaling, altering dopaminergic, glutamatergic, GABAergic, and purinergic systems, and inducing maladaptive changes in the phase and amplitude of circadian oscillations. This may create phasic interference between oscillatory neurotransmitter systems and thereby promote pathologic changes in synaptic plasticity and behavior. Such magnitude of global disruption may make it unfeasible to target any single receptor or neurotransmitter system in order to effectively treat AUD, perhaps justifying the use of medications or treatments which more broadly affect diverse signaling systems or the circadian clock. This logic has proven moderately useful for treatment of severe depression, which is significantly although transiently mitigated by sleep deprivation therapy (Dopierała and Rybakowski, 2015). Similar treatment options targeting broad behavioral patterns of sleep and activity or largely interrupting or arresting circadian and non-circadian rhythms of multiple forms of neurotransmission may act as effective “neurologic defibrillators” that cease maladaptive or misaligned neurochemical oscillations and permit the restoration of healthy rhythms of neurotransmission and behavior. Alternatively, specific targeting of adenosine or other neurotransmitters that are highly integrated with multiple signaling systems and affect both neuronal and astrocytic function may effectively reset and realign molecular and signaling rhythms adversely affected in AUD. Moreover, the efficacy of these targeted or untargeted treatments may be dependent upon the time of administration. This is because any targeted therapy may have variable affects depending on the current phase of the targeted signaling pathway. This is especially true for signaling systems that are highly dependent upon the circadian clock such as ATP and adenosine. Thus, experimental treatment options for AUD must take into consideration the extraordinary integration of purinergic signaling with neuronal and astrocytic function as well as the time-dependent oscillations of these signaling systems and aim to mitigate the robust and all-consuming cycles of neurotransmission and behavioral cravings that underlie AUD.

## Author contributions

DL, LA-B, Y-FJ, SK, and D-SC wrote the manuscript. DL and SK prepared figures. D-SC reviewed and edited the manuscript.

### Conflict of interest statement

The authors declare that the research was conducted in the absence of any commercial or financial relationships that could be construed as a potential conflict of interest.
